# Multinomial network meta-analysis using response rates: relapsed/refractory multiple myeloma treatment rankings differ depending on the choice of outcome

**DOI:** 10.1186/s12885-022-09571-8

**Published:** 2022-05-30

**Authors:** Chrissy H. Y. van Beurden-Tan, Pieter Sonneveld, Carin A. Uyl-de Groot

**Affiliations:** 1 Erasmus MC Cancer Institute, Rotterdam, The Netherlands; 2 Erasmus School of Health Policy & Management /Institute for Medical Technology Assessment, Erasmus University Rotterdam, The Netherlands Rotterdam,

**Keywords:** Multiple myeloma, Network meta-analysis, Response outcomes, Treatment ranking, SUCRA

## Abstract

**Background:**

Due to the fast growing relapsed/refractory multiple myeloma (RRMM) treatment landscape, a comparison of all the available treatments was warranted. For clinical practice it is important to consider both immediate effects such as response quality and prolonged benefits such as progression-free survival (PFS) in a meta-analysis. The objective of this study was to assess the impact of the choice of outcome on the treatment rankings in RRMM.

**Methods:**

A multinomial logistic network meta-analysis was conducted to estimate the ranking of sixteen treatments based on both complete and objective response rates (CRR and ORR). Seventeen phase III randomized controlled trials from a previously performed systematic literature review were included. Treatment ranking was based on the surface under the cumulative ranking curve (SUCRA). Sensitivity analysis was conducted.

**Results:**

The ranking of treatments differed when comparing PFS hazard ratios rankings with rankings based on CRR. Pomalidomide, bortezomib and dexamethasone ranked highest, while a substantial lower ranking was observed for the triplet elotuzumab, lenalidomide, dexamethasone. The ranking of treatments did not differ when comparing PFS hazard ratios and ORR. The scenario analyses showed that the results were robust. In all scenarios the top three was dominated by the same triplets. The treatment with the highest probability of having the best PFS and ORR was the triplet daratumumab, lenalidomide plus dexamethasone in the base case.

**Conclusion:**

This analysis shows that depending on the chosen outcome treatment rankings in RRMM may differ. When conducting NMAs, the response rate, a clinically recognized outcome, should therefore be more frequently considered.

**Supplementary Information:**

The online version contains supplementary material available at 10.1186/s12885-022-09571-8.

## Background

Many treatment combinations are currently available for treating relapsed and/or refractory multiple myeloma (RRMM) patients and more will be added in rapid pace [1]. Current treatments are mostly combinations of the first-generation proteasome inhibitor (PI) bortezomib or the immunomodulatory drug (IMiD) lenalidomide, in combination with the second-generation PIs (carfilzomib, ixazomib), iMiD ( pomalidomide), HDAC inhibitors (vorinostat, panobinostat), monoclonal antibodies (elotuzumab, daratumumab, isatuximab) and three miscellaneous drugs (dexamethasone, oblimersen, pegylated liposomal doxorubicin).

For cross-trial comparisons, a network meta-analysis (NMA) using binomial outcomes is the most common way of synthesizing available treatment evidence. This synthesized treatment evidence is relevant for doctors and patients, but also for HTA bodies like the National Institute for Health and Care Excellence (NICE) [2]. Several NMAs have already been conducted in this target patient population comparing available MM treatments using progression-free survival (PFS). The first NMA including all available RRMM phase III randomized controlled trials (RCTs) was published in 2017, which compared PFS hazard ratios of seventeen published trials [3]. Eight other full-text publications [4–11] were found presenting NMAs in RRMM, which all used a binomial approach and applied restrictions on the number of trials. All these NMAs in RRMM used binomial outcomes like odds ratios of complete or objective response rates, HRs for survival outcomes as PFS and OS or risk ratios for adverse events.

Although binomial outcomes has shown to be broadly applicable for different types of clinical outcomes a multinomial NMA would better represent the response outcome. Response rates being an important outcome for MM patients, often cover more than two categories, therefore synthesizing this outcome would need more adjustments if the method is limited to binomial outcomes. For example, in case of three categories A, B and C the binomial model could be used in which first the odds ratio of A versus “not A” (i.e., B and C) is synthesized. Then as a second nested step the odds ratio of B versus C could be synthesized using the traditional binomial approach. After obtaining all the odds ratios the actual estimated rates per category can be obtained. The multinomial NMA makes the intermediate step obsolete and is designed to synthesize clinical outcomes that cover more than two categories.

Therefore, the objective of this study is to explore the difference in outcomes between a binomial NMA and a multinomial NMA in RRMM. The multinomial NMA included the different response rates per response category (i.e. complete response (CR), partial response (PR) and less than PR (< PR)).

## Methods

### Systematic literature review

The systematic literature review was previously described in detail [3]. In short, studies were included if they described a phase III RCT among adult patients with RRMM. Furthermore, the regimens of the RCT had to include at least one of the prespecified novel treatments. Several literature databases were searched within a timeframe from 01 January 1999 to 01 April 2020.

### Data extraction

Data was extracted from phase III RCTs that reported the number or the proportion of patients achieving an objective response. A thorough description of the whole data extraction process was described before [3]. The responses were grouped into three categories: Complete Response or better (CR), Partial Response (PR) and < PR. The CR-group contained CR, stringent CR (sCR) and near CR (nCR). The PR-group consisted of very good PR (VGPR) and PR, and in the < PR-group the remaining categories like minimal response (MR), no change (NC), and progressive disease (PD) were grouped. In case there were zero responders in at least one category within an RCT, a zero-correction factor of k = 1 was added to all the fields in the data table of that specific trial to properly run the NMA [12].

### Network meta-analysis

A Bayesian multinomial logistic model was built based on a competing risk NMA model, described by Ades et al. [12] and published in the NICE DSU Technical Support Document 2 [13]. The NMA is an iterative process. Each iteration was based on a different set of patients per response category for each trial and arm. The number of patients per response category were drawn from the 95% confidence intervals (CIs) of a Multinomial distribution. After all simulations, the NMA calculated the probability that the treatment ranked a certain rank *k*. These probabilities were then used to calculate the surface under the cumulative ranking (SUCRA). The SUCRA can range between 0 and 100%. When SUCRA is close to 100%, the treatment is very likely to be the best treatment and when it is close to 0%, the treatment is very likely to rank last. Results based on objective response rates (defined as responses ≥ PR and abbreviated as ORR) were also considered in this NMA defined as ORR SUCRA. Appendix A provides an illustrative numerical example of the NMA method applied in this research.

Since the NMA method is an iterative process using different sets of patients distributed over the three response categories based on their 95% CIs, it is important to determine convergence of the results. This was assessed using the Brooks-Gelman-Ruben diagnostic *R* which is available in WinBUGS. This software provides a graphical representation of this convergence diagnostic and shows the diagnostic after each iteration of the different sets of patients in which one should be concerned both with convergence of *R* to 1, and with convergence of both the pooled and within interval widths to stability.

Another significant check is the face-validity of the NMA outcomes. Face-validity was checked by comparing the computed response rates by the NMA with the response rates reported in the publications of the trials.

### Scenario analyses

Three scenario analyses were conducted to test the robustness of the study results: [1] the MM-003 trial was removed from the NMA since the patient population is heavily pretreated in comparison with the other included trials, [2] the doublet lenalidomide plus dexamethasone was used as the NMA reference treatment instead of dexamethasone to see the impact on the results and [3] trials with at least one zero event (i.e. for CR) were removed (i.e. MM-003[14], GMY302[15] and Hjorth2012[16]).

## Results

### Systematic literature review

A detailed description and PRISMA flow chart of the systematic literature review can be found in Van Beurden-Tan et al. [3]. Of the total 19,773 citations retrieved from the different databases and the two added abstracts, 71 citations were eligible for full text analysis. These citations included sixteen RCTs of which fifteen full publications and one conference abstract. Another RCT was identified through screening ClinicalTrials.gov and resulting in a total of seventeen identified phase III RCTs. The details of the RCTs are shown in Table 1.

**Table 1:** Details and data extraction of relapsed and/or refractory multiple myeloma phase III RCTs

Trial ID NCT number	Treatment (control versus experimental)	Median age (range)	Median prior lines (range)	N itt	# CR-group	# PR-group	# < PR-group	Duration
GMY302 NCT00017602	Dexamethasone Oblimersen + Dexamethasone	65 (n/r-n/r) 59 (n/r-n/r)	3 3	114 110	0 0	19 16	95 94	Dec 2000—Apr 2009
APEX NCT00048230	Dexamethasone Bortezomib	61 (47–73) 62 (48–74)	2 2	336 333	5 41	51 80	280 212	Jun 2002—Dec 2004
MM-009 NCT00056160	Dexamethasone Lenalidomide + Dexamethasone	62 (37–85) 64 (38–86)	n/r n/r	176 177	3 43	32 65	141 69	Jan 2003—Oct 2008
MM-010 NCT00424047	Dexamethasone Lenalidomide + Dexamethasone	64 (40–82) 63 (33–84)	2 2	175 176	9 43	33 63	133 70	Sep 2003—Nov 2013
Orlowski NCT00103506	Bortezomib Pegylated Liposomal Doxorubicin + Bortezomib	62 (34–88) 61 (28–85)	n/r n/r	322 324	8 14	125 130	189 180	Dec 2004—Jun 2014
Garderet 2012 NCT00256776	Thalidomide + Dexamethasone Bortezomib + Thalidomide + Dexamethasone	63 (39–75) 60 (29–76)	n/r n/r	134 135	25 56	61 52	48 27	Jul 2005—Jun 2013
OPTIMUM NCT00452569	Dexamethasone Thalidomide^t^	63 (40–86) 63 (33–86)	n/r (1–3) n/r (1–3)	126 373	2 7	23 53	101 313	Feb 2006—Jan 2009
Hjorth 2012 NCT00602511	Bortezomib + Dexamethasone Thalidomide + Dexamethasone	71 (50–84) 71 (38–85)	n/r n/r	64 67	0 0	40 37	24 30	Oct 2007—Dec 2010
OPTIMISMM NCT0173492	Bortezomib + Dexamethasone Pomalidomide + Bortezomib + Dexamethasone	68 (59–73) 67 (60–73)	2 (1–2) 2 (1–2)	278 281	11 44	128 187	139 50	Jan 2013—May 2017
PANORAMA1 NCT01023308	Bortezomib + Dexamethasone Panobinostat + Bortezomib + Dexamethasone	63 (56–68) 63 (56–69)	2 (1–3) 2 (1–3)	381 387	60 107	148 128	173 152	Dec 2009—Jul 2015
ASPIRE NCT01080391	Lenalidomide + Dexamethasone Carfilzomib + Lenalidomide + Dexamethasone	65 (31–91) 64 (38–87)	2 (1–3) 2 (1–3)	396 396	37 126	227 219	132 51	Jul 2010—Oct 2017
MM-003 NCT01311687	Dexamethasone Pomalidomide + Dexamethasone	65 (35–87) 64 (35–84)	5 (2–17) 5 (2–14)	153 302	0 3	15 92	138 207	Mar 2011—Sep 2017
ELOQUENT-2 NCT01239797	Lenalidomide + Dexamethasone Elotuzumab + Lenalidomide + Dexamethasone	66 (38–91) 67 (37–88)	2 (1–4) 2 (1–4)	325 321	24 14	189 238	112 69	Mar 2011—Mar 2018
ENDEAVOR NCT01568866	Bortezomib + Dexamethasone Carfilzomib + Dexamethasone	65 (30–88) 65 (35–89)	2 (1–3) 2 (1–3)	465 464	29 58	261 298	175 108	Jun 2012—Dec 2018
Tourmaline-MM1 NCT01564537	Lenalidomide + Dexamethasone Ixazomib + Lenalidomide + Dexamethasone	66 (30–89) 66 (38–91)	n/r (1–3) n/r (1–3)	362 360	24 42	235 240	103 78	Aug 2012—Dec 2020
Pollux NCT02076009	Lenalidomide + Dexamethasone Daratumumab + Lenalidomide + Dexamethasone	65 (42–87) 65 (34–89)	1 (1–8) 1 (1–11)	283 286	54 123	162 143	67 20	May 2014—Sep 2020
Castor NCT02136134	Bortezomib + Dexamethasone Daratumumab + Bortezomib + Dexamethasone	64 (33–85) 64 (30–88)	2 (1–10) 2 (1–9)	247 251	21 46	127 153	99 52	Aug 2014—Mar 2017

*n/r* not reported, *t *this was a four-arm study with three different dosing Thalidomide arms (i.e. 100 mg, 200 mg, and 400 mg) combined in one arm for this research

In these RCTs, eighteen different treatment options were used. For this analysis the addition of dexamethasone to bortezomib or thalidomide is assumed to have equal efficacy results as bortezomib or thalidomide monotherapy. Therefore, instead of having eighteen treatment options, sixteen treatment options were included in the current NMA. Because vorinostat does not have regulatory approval for myeloma therapy, the VANTAGE-088 trial was omitted from this analysis [17].

### Data extraction

The extracted data is presented in Table 1. The oldest included trial started in the year 2000 and the median follow-up ranged from 5·59 months (PANORAMA1 [18]) to 32·3 months (ASPIRE [19]). The age ranged from 28 to 91 years, with the median age above 59 years. The response criteria evolved over time; therefore seven types of response definitions [20–26] were found among which the EBMT [21] (older studies), Richardson [26] (for nCR) and IMWG [22] (most recent studies) were mostly used. In all those response definitions CR and PR remained the same, but the changes were in the additional categories like stringent CR and VGPR. The number of previous therapies were comparable with a median of 2 prior therapies, except for the MM-003 pomalidomide trial [14] which included patients with a median of five prior lines of therapies. In total 9080 patients were included in this NMA. The smallest study enrolled 131 patients (Hjorth 2012 [16]) and the largest enrolled 768 patients (PANORAMA1 [18]).

The number of patients in the defined three response categories were extracted for the different studies. The details are shown in the columns # CR-group, # PR-group and # < PR-group respectively of Table 1. In total, 1079 patients in the CR-group, 4070 in the PR-group and 3931 in the < PR-group were included. In one study (OPTIMUM [27]) the responses were presented in rates only, while all the others presented them in both rates and numbers. In three studies [14–16] there were no patients in the CR-group. For these studies, a zero-correction factor of 1 has been applied to run the NMA. The complete response rates of dexamethasone alone ranged from 0% (GMY302 [15] and MM-003[14]) to 5% (MM-010 [28]), while lenalidomide plus dexamethasone ranged from 7% (in ELOQUENT-2 [29] and Tourmaline-MM1[30]) to 24% (in MM-009 [31] and MM-010 [28]) and of bortezomib with or without dexamethasone ranged from 0% (in Hjörth 2012 [16]) to 16% (in PANORAMA1 [18]).

### Network meta-analysis

**Fig. 1:**
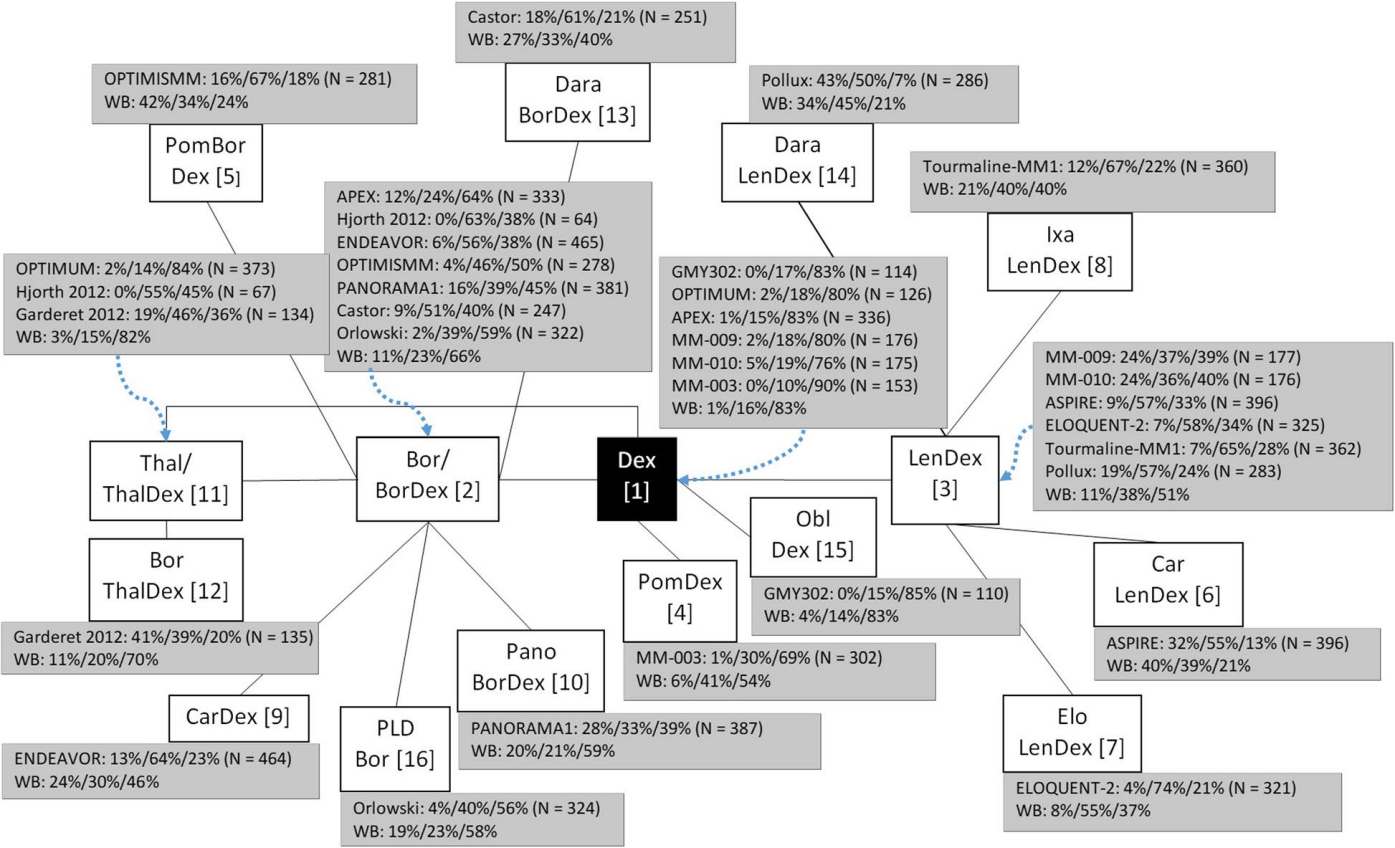
Network of relapsed/refractory MM RCTs used for the multinomial network meta-analysis based on response. Legend: format of dark grey results box = `RCT name’: %CR/%PR/% < PR (*N* = `total number of patients’), * indicates estimated from other values, and black box indicates the reference treatment. Abbreviations: *WB* WinBUGS, NMA result, *Bo* bortezomib, *Dex* dexamethasone, *Tha* thalidomide, *Car* carfilzomib, *Obl* oblimersen, *Dara* daratumumab, *Len* lenalidomide, *Pom* pomalidomide, *Ixa* ixazomib, *Elo* elotuzumab, *Pano* panobinostat, *Vorino* = vorinostat, *PLD* pegylated liposomal doxorubicin

Figure 1 presents the complete network for RRMM treatments. We assumed that: i) the relative efficacy of bortezomib [26, 32] versus dexamethasone is identical to bortezomib plus dexamethasone [16, 18, 33–35] versus dexamethasone, ii) the relative efficacy of thalidomide [27] versus dexamethasone is identical to thalidomide plus dexamethasone [16, 36] versus dexamethasone, iii) no difference in efficacy due to administration method (intravenous [16, 18, 26, 32] versus subcutaneous [34, 35] bortezomib) and dosage scheme (100 versus 200 versus 400 mg thalidomide [27]). Consequently, the three thalidomide treatment arms (i.e. 100 mg, 200 mg and 400 mg thalidomide) in the OPTIMUM trial [27] were pooled by summing up the number of patients in the three thalidomide arms.

The WinBUGS code to run the full NMA is presented in Appendix B. Three different chains were simulated. Each chain produced 25,000 iterations as burn-in samples and the following 80,000 were used for parameter estimations. Inspection of the Brooks-Gelman-Rubin plots showed convergence of the model parameters within these samples.

### Complete response rates

**Fig. 2:**
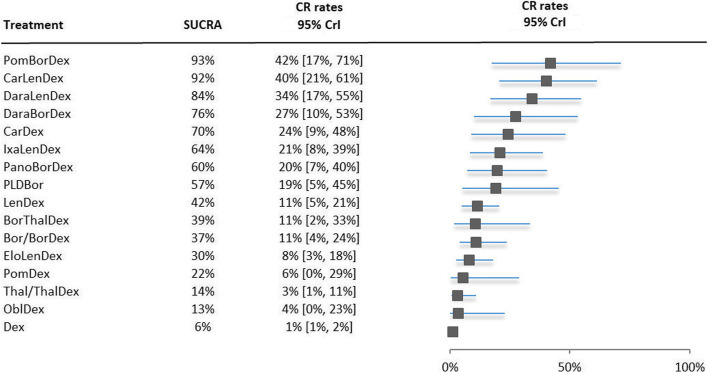
Forest plot of NMA results proportion of complete response patients. The treatments are ranked from highest to lowest, with the best treatment on top. Abbreviations: *Bor* bortezomib, *Car* carfilzomib, *Dara* daratumumab, *Dex* dexamethasone, *Elo* elotuzumab, *Ixa* ixazomib, *Len* lenalidomide, *Obl* oblimersen, *Pano* panobinostat, *PLD* pegylated liposomal doxorubicin, *Pom* pomalidomide, *Thal* thalidomide, *Vorino* vorinostat

Figure 2 presents the NMA results in which dexamethasone was used as reference treatment in a forest plot. All treatments were sorted based on their SUCRA ranking and accompanied by their CRR with the 95% Credible Intervals (CrIs). The distribution of the probabilities of being at each rank, together with the mean rank for CRR are presented in Appendix C.

Pomalidomide, bortezomib plus dexamethasone and carfilzomib, lenalidomide plus dexamethasone dominated the top of the ranking in the forest plot indicating the best treatments with regards to CRRs in RRMM. The triplet pomalidomide, bortezomib plus dexamethasone was identified as the treatment with on average the highest CRR, while the triplet carfilzomib, lenalidomide plus dexamethasone had a narrower CrI indicating less uncertainty around the CRR point estimate. However, all 95% CrIs were wide, and a lot of the CrIs overlapped with other treatments. Four treatments, i.e. 1: lenalidomide plus dexamethasone, 2: elotuzumab, lenalidomide plus dexamethasone, 3: thalidomide with or without dexamethasone, and 4: dexamethasone mono therapy had no overlapping CrIs with carfilzomib, lenalidomide plus dexamethasone.

In the POLLUX trial a significant difference in PFS HR between daratumumab, lenalidomide plus dexamethasone versus lenalidomide plus dexamethasone was observed and a significant difference in CRR was expected as well. However, in the NMA results the 95% CrI of the triplet overlaps with the doublet lenalidomide plus dexamethasone’s with respect to the CRR outcome.

The older regimens were at the bottom of the forest plot indicating being the less effective treatments with regards to CRRs. However, also the newer elotuzumab, lenalidomide plus dexamethasone triplet is ranked in the bottom half.

### Objective response rates

**Fig. 3:**
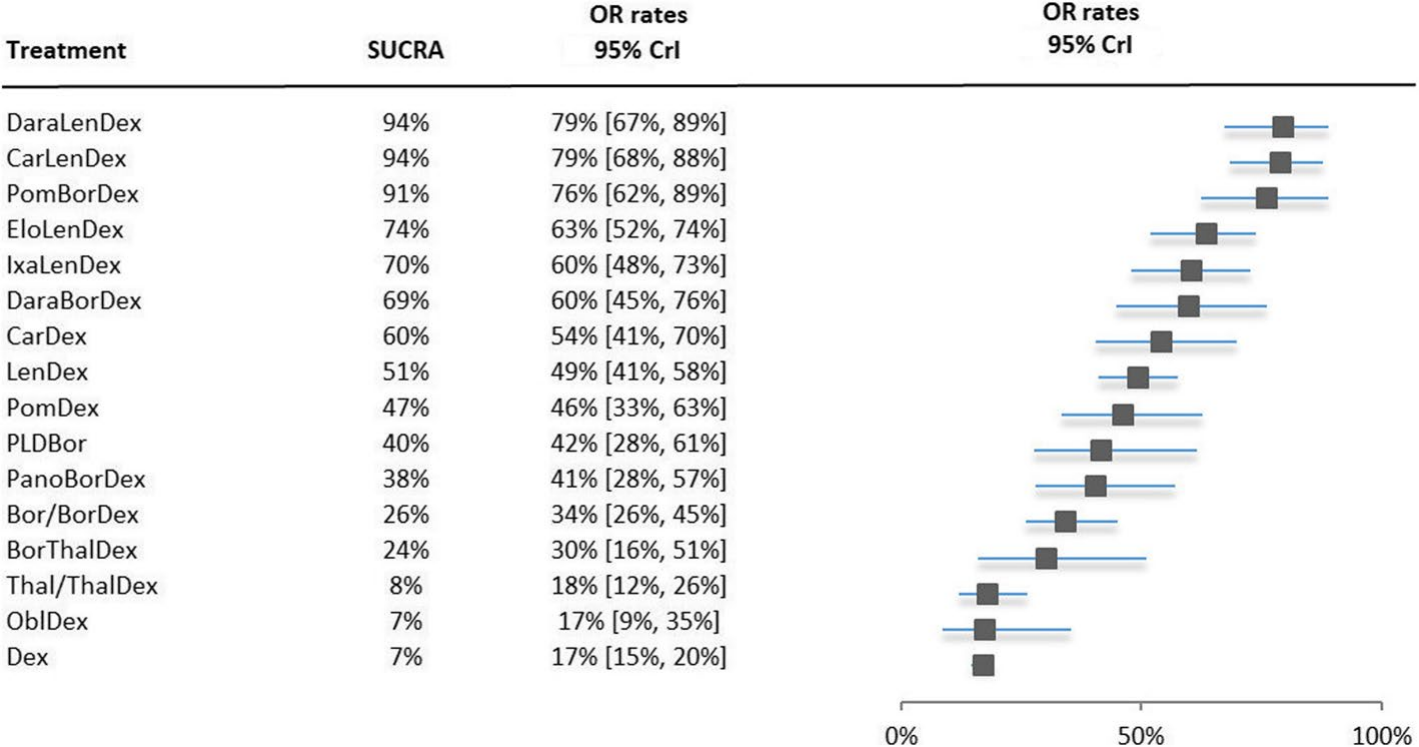
Forest plot of NMA results proportion of objective response patients. The treatments are ranked from highest to lowest, with the best treatment on top. Abbreviations: *Bor* bortezomib, *Car* carfilzomib, *Dara* daratumumab, *Dex* dexamethasone, *Elo* elotuzumab, *Ixa* ixazomib, *Len* lenalidomide, *Obl* oblimersen, *Pano* panobinostat, *PLD* pegylated liposomal doxorubicin, *Pom* pomalidomide, *Thal* thalidomide, *Vorino* vorinostat

Figure 3 presents the NMA results based on the ORRs. All treatments were ranked on their SUCRA ranking and accompanied by their ORR with the 95% CrI. The distribution of the probabilities of being at each rank, together with the mean rank for ORR and the SUCRA curve are presented in Appendix C.

The treatment ranking changed slightly for the majority of the treatments when comparing the ranking based on ORR with those seen in CRR. The triplet daratumumab, lenalidomide plus dexamethasone moved from third rank to first. However, this triplet tied with the triplet carfilzomib, lenalidomide plus dexamethasone with an average of 79% ORRs. Daratumumab, lenalidomide plus dexamethasone is now ranked first and identified as the treatment with on average the highest ORR with slightly narrower CrI. Pomalidomide, bortezomib plus dexamethasone took the third rank compared to first in CRR results. The CrI of nine treatments did not overlap with the top 2 triplets. Elotuzumab, lenalidomide plus dexamethasone triplet is ranked 4^th^ in the ORR results opposed to the 12^th^ position in the CRR results.

### Comparing PFS HR results with response rates results

The Van Beurden-Tan et al. [3] network (PFS NMA ranking) has been updated to match the present network in Fig. 1 and to enable a comparison between the two NMAs on SUCRA. The VANTAGE-88 (vorinostat) trial was deemed irrelevant and therefore removed from the network, while the OPTIMISMM (pomalidomide, bortezomib plus dexamethasone) trial was included as a highly relevant new trial that was not included in the previously conducted PFS NMA. The distribution of the probabilities of being at each rank, together with the mean rank for PFS and the SUCRA curve are presented in Appendix C.

**Fig. 4:**
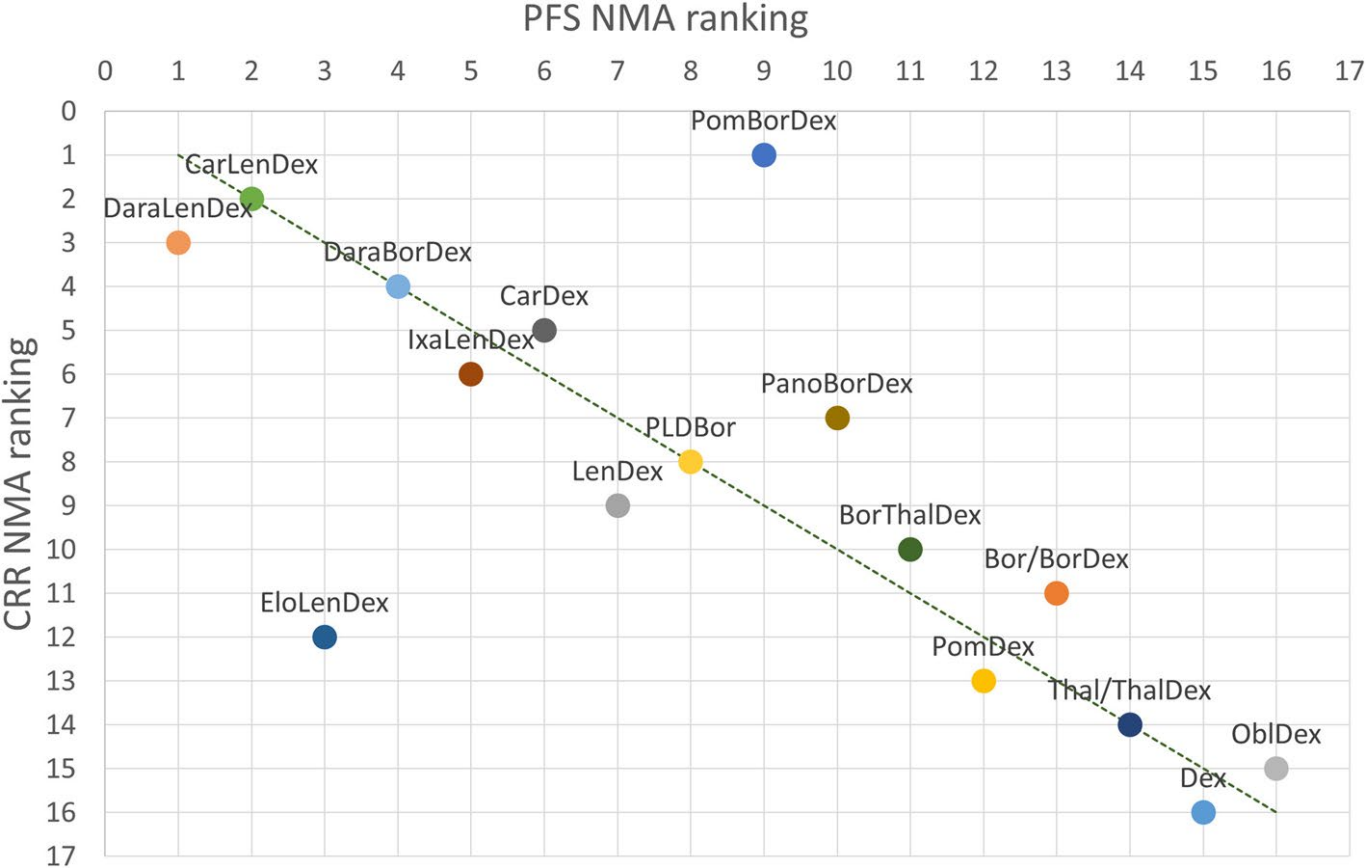
CRR ranking versus PFS ranking. Comparing the ranking of RRMM treatment combinations of binomial NMA of PFS HR NMA versus multinomial NMA of CRR

**Fig. 5:**
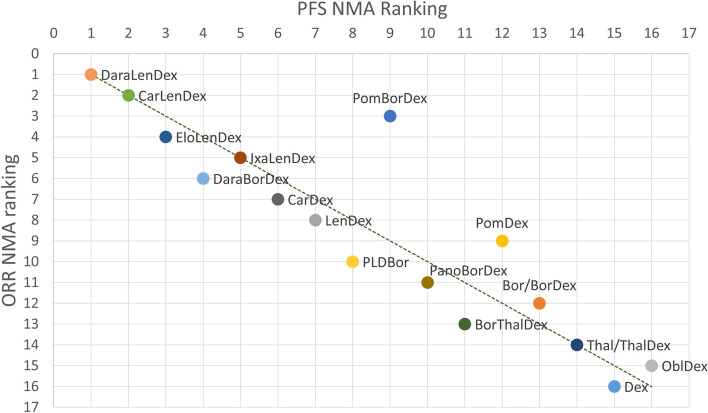
ORR ranking versus PFS ranking. Comparing the ranking of RRMM treatment combinations of binomial NMA of PFS HR NMA versus multinomial NMA of ORR

Figure 4 and Fig. 5 are scatterplots showing the relationship between the PFS NMA SUCRA ranking and the CRR and ORR SUCRA rankings respectively. For every treatment combination the PFS NMA ranking is plotted on the x-axis and the response ranking is plotted on the y-axis: CRR in Fig. 4 and ORR in Fig. 5. A dotted trend line is drawn in both figures indicating perfect alignment of the PFS and response ranking when points resided on this line in the graph.

Pomalidomide, bortezomib plus dexamethasone ranked 9^th^ in the PFS HR NMA opposed to 3^rd^ in ORRs ranking and even 1^st^ in CRR. In addition, elotuzumab, lenalidomide plus dexamethasone appeared to be an outlier in the CRRs results with a big drop in ranking. Some other shifts of rankings were visible in the bottom ranks; however, these were small.

### Scenario analyses

The scenario analyses results are presented in the Supplemental material. In none of the scenarios the order of treatment ranking changed. When the doublet lenalidomide plus dexamethasone was selected as reference treatment (scenario 2) in the NMA the width of the credible intervals for lenalidomide-based treatments decreased (implying less uncertainties around the point estimates) while those of bortezomib-based (Appendix D) increased. Although treatment ranking order did not change in the scenarios, the absolute values did change, and the biggest change was seen in scenario 4 when the RCTs with zero events were removed from the network (Appendix D). The highest absolute CRR was seen in scenario 3 (51% versus 42% in base case), while the highest absolute ORR was seen in scenario 2 (88% versus 79% in base case).

## Discussion

With the ever-evolving RRMM treatment playing field, it is essential to help doctors and others involved in the choice of treatment for patients by quantifying all available data as best we can. There are many treatment choices at first relapse in MM and the choice and sequence depends also on prior drug exposures, drug-refractoriness, patient comorbidities, and high-risk cytogenetics, among others. However, the first step in helping is getting an overall sense of the on average best treatment and how all treatments compare against each other. It is common practice to synthesize the available efficacy evidence in a binomial Bayesian NMA. Common outcomes for such NMAs are HRs for survival outcomes (i.e. PFS, time to progression and OS), or odds ratios for response (CRR versus < CRR or response versus no response). However, since response is categorized in more than two categories (i.e., non-binomial), it resulted in the question whether it would make sense to synthesize the data in a multinomial NMA.

Our study used seventeen phase III RCTs resulting in sixteen RRMM treatment regimens using a Bayesian multinomial logistic NMA calculating three response rates categories: CR, PR and < PR. This NMA showed it is possible to deviate from the classical binomial NMA with PFS HRs. Treatments were either compared on CRRs or ORRs (i.e. ≥ PR). The triplet pomalidomide, bortezomib plus dexamethasone was the best treatment option when considering CRR in contrast to the triplet daratumumab, lenalidomide plus dexamethasone when ORR was the main outcome parameter. However, the credible intervals of these two regimens overlap and therefore interpretation of the results should be done with caution.

To our knowledge, this study is the first to present a multinomial NMA on response in RRMM. One study by Botta et al. [9] presented overall survival (OS) HRs, odds ratios of complete response (CR), odds ratios of objective response (OR), and relative risk ratios of adverse events. They also included three phase II RCTs [37–39] in order to include siltuximab plus bortezomib, bortezomib plus bevacizumab and elotuzumab, bortezomib plus dexamethasone in their network. They concluded IMiDs plus new anti-MM monoclonal antibodies-containing regimens (i.e. lenalidomide plus dexamethasone in combination with daratumumab or elotuzumab) were the best therapeutic options in RRMM. Another study by Luo et al. [10] extended the Van Beurden-Tan et al. NMA by including the three treatments from the phase II RCTs (which were included in Botta et al. [9]), and compared all treatment combinations on nonresponse rate (NRR) odds ratios, time to progression HR, PFS HR and OS HR. The triplet combination daratumumab, lenalidomide plus dexamethasone remained the overall best treatment option, however the triplet ixazomib, lenalidomide plus dexamethasone had the best OS efficacy according to their results. Other NMA studies did not include response rates and none of the studies used multinomial logistic NMAs for the response outcome.

This study’s results were similar to the results presented by Botta et al. [9]. A direct comparison of the SUCRA ranks of their CR and ORR NMAs is due to the different included treatments therefore not directly feasible. However, similar trends in ranks were shown; having the triplets daratumumab, lenalidomide plus dexamethasone and carfilzomib, lenalidomide plus dexamethasone in the top ranking and the older doublet regimens at the bottom. The triplet elotuzumab, lenalidomide plus dexamethasone rank was also substantially lower in their CR outcome than compared to their ORR NMA.

The most obvious strength of our study is the practical exercise of conducting a different type of NMA for RRMM treatments. Other researchers can use this study to perform a multinomial NMA in for instance other hematological cancers. It can also encourage others to perform a multinomial NMA additionally to the binomial NMAs already published to compare the difference in conclusions using a different NMA method.

Although every NMA has limitations with respect to comparability of included trials, our study aimed to show the impact of selecting a different NMA type (multinomial versus binomial). Two list of rankings (one based on CRR and the other on ORR) were compared to the ranking based on PFS HRs presented previously [3]. First, this study’s ORR ranking was very similar to the PFS HR ranking with pomalidomide, bortezomib plus dexamethasone as an exception. Second, the CRR ranking showed more differences when compared to the PFS HR rankings. In the CRR ranking the best treatment option was pomalidomide, bortezomib plus dexamethasone and a lot of movements in ranks were seen partly explained by the lower ranking for the triplet elotuzumab, lenalidomide plus dexamethasone. This lower ranking was expected as this triplet also resulted in a substantially lower CRR in the ELOQUENT-2 trial. The lower percentage of CR or higher in patients treated with elotuzumab, lenalidomide, dexamethasone may at least be partly explained by interference of the antibody with serum protein electrophoresis and/or immunofixation, which may result in false-positive results. Therefore, the true CR rate of elotuzumab, lenalidomide plus dexamethasone may have been higher.

Considering limitations for the current study, the most important assumption was assigning a similar response profile for bortezomib with or without dexamethasone (and for consistency also for thalidomide with or without dexamethasone). The assumption that the relative efficacy of bortezomib monotherapy and the doublet bortezomib plus dexamethasone being similar, has been often challenged by other researchers. This assumption was made by Van Beurden-Tan et al. to bridge the bortezomib-based regimens with the lenalidomide-based regimens to obtain a singular network containing all RRMM regimens [3]. This assumption was justified by the findings by Ludwig et al. [40] that elderly RRMM patients should be administered dexamethasone at a lower dose and for a shorter duration of time because of a statistically significant increase in early deaths. It was decided not to change the network by adding observational studies [11], because of uncertain quality of data. Thus, the results of this NMA could be compared with our previous NMA [3] after updating the original network by removing the VANTAGE-88 [41] and adding the OPTIMISMM [33] trial.

Another limitation of our study lies in the choice for three response categories. The IMWG response criteria [22] listed more than three response categories. However, in this study it was decided to group all available responses into three categories. First, additional response grading was added with the introduction of the IMWG response criteria which were not available before [22]. Secondly, since the aim was to conduct a multinomial NMA we required more than two outcome categories. It was decided to use three to have enough granularity to show that CR-group and PR-group patients might be different. Using more than three outcome categories would have resulted in too low number of patients per category.

Very important to mention is the significant heterogeneity in the patient populations, particularly in relation to exposure and refractory status to specific agents as the trials included span over 15 years in time. Some patients were never exposed to the newer agents that entered the RRMM treatment paradigm later in time.

We also expect that in the future the multinomial NMA might be used in synthesizing data on MRD (minimal residual disease) status in combination with classical responses. A classification based on MRD status (i.e. negative versus positive) in combination with the classical responses (e.g. MRD-negativity [MRD(-)] in VGPR patients [42]) can result in more than two outcome categories (i.e. MRD(-) in CR, MRD( +) in CR, MRD(-) in VGPR, MRD( +) in VGPR). A multinomial NMA can support this type of outcome as it supports more than two outcome categories.

Not only do physicians have to decide which treatment combination is the best, with the ever-pressing health care budgets there is also a high need to choose the most optimal treatment in terms of cost-effectiveness. Carlson et al. investigated the cost-effectiveness of RRMM treatment regimens in the US [7]. They concluded that only the addition of daratumumab or panobinostat may be considered cost-effective options according to commonly cited thresholds. It would therefore be interesting to quantify the impact of the choice of NMA type (binomial versus multinomial) and/or different outcomes (PFS/OS versus response rates) on the conclusion of a cost-effectiveness analysis. Since we did not find a big difference in results between the PFS and ORR NMAs, we also do not expect a major difference in cost-effectiveness results based on one of these two. However, some prominent differences were seen between the PFS and CRR NMA results, therefore differences in cost-effectiveness results may be expected, and should therefore be investigated in future research.

## Conclusion

This research showed that NMAs based on ORRs and PFS HRs resulted in similar treatment rankings of RRMM treatments in terms of efficacy. Differences in treatment rankings were only observed when comparing the ranking results of the PFS NMA with those from the CRRs NMA and was only driven by the lower ranking position of the elotuzumab triplet.

Therefore, selection of NMA type and outcome should be done sensibly since this might a priori influence the direction of the cost-effectiveness results and decision makers should be aware of the possible differences and therefore consequences when selecting NMA type and outcomes.

## Supplementary Information

**Additional file 1:** **Appendix A.** Multinomial network meta-analysis WinBUGS code, init and data files

**Additional file 2:** **Appendix B.** Detailed description and numerical example of multinomial network meta-analysis 

**Additional file 3:** **Appendix C.** SUCRA results

**Additional file 4: Appendix D. **Results scenario analyses

## Data Availability

All data generated or analysed during this study are included in this published article (and its supplementary information files).
