# Supplementary material for: Multinomial network meta-analysis using response rates: relapsed/refractory multiple myeloma treatment rankings differ depending on the choice of outcome
Source: BMC Cancer. 2022 May 30;22:591. doi: 10.1186/s12885-022-09571-8 (PMC9150316; doi:10.1186/s12885-022-09571-8)
Supplement: Supplementary file 1 — Additional file 1: Appendix A. Multinomial network meta-analysis WinBUGS code, init and data files [file 12885_2022_9571_MOESM1_ESM.docx]

# Appendix A

# Multinomial network meta-analysis WinBUGS code, init and data files

This appendix contains all the WinBUGS codes that were used to run the multinomial network meta-analysis in RR MM treatments using response outcomes.

# Start script

# Multinomial likelihood

# Fixed effect model

model{ # *** PROGRAM STARTS

for(i in 1:ns){ # LOOP THROUGH STUDIES

for(k in 2:ne){ # LOOP OVER 2 ENDPOINTS

a[i,k] ~ dnorm(0,.001) # vague priors for all trial baselines

}

for(j in 1:na[i]){ # LOOP THROUGH ARMS

r[i,j,1:ne]~dmulti(p[i,j,1:ne],n[i,j]) # MULTINOMIAL LIKELIHOOD

p[i,j,1]<- 1-sum(p[i,j,2:ne])

slam[i,j] <- sum(lambda[i,j,2:ne]) # sum of the two hazards

for(k in 1:ne){ # LOOP OVER ALL ENDPOINTS

rhat[i,j,k] <- p[i,j,k]*n[i,j] # predicted number of events

dv[i,j,k] <- 2*r[i,j,k]*log(r[i,j,k]/rhat[i,j,k]) # deviance contribution

}

dev[i,j] <- sum(dv[i,j,]) # deviance contribution for arms

for(k in 2:ne){ # LOOP THROUGH END POINTS

p[i,j,k] <- lambda[i,j,k]/(1+slam[i,j]) # probability of end point k for study i arm j

log(lambda[i,j,k]) <- a[i,k] + d[Tx[i,j],k] - d[Tx[i,1],k] # model for linear predictor

}

}

resdev[i] <- sum(dev[i,1:na[i]]) # summed residual deviance contribution for this trial

}

totresdev <- sum(resdev[]) # Total residual deviance

for(k in 2:ne){ # LOOP THROUGH ALL ENDPOINTS

d[1,k] <- 0 # treatment effect is zero for reference treatment

for(t in 2:nt){ d[t,k] ~ dnorm(0,.001) } # vague priors for treatment effects

}

for(k in 2:ne){ # LOOP THROUGH 2 ENDPOINTS

a_av[k] <- mean( a[1:ns1,k] ) # pooled mean average of studies containing reference tx

}

p_av[1,1] <- 1/(1+(exp(a_av[2]) + (exp(a_av[3])))) # pooled probability reference treatment first endpoint

for(k in 2:ne){ # LOOP TROUGH END POINTS

p_av[1,k] <- p_av[1,1]*exp(a_av[k]) # pooled probabilities refrefreference treatment remaining endpoints

}

for(t in 2:nt){ # LOOP OVER TREATMENTS

p_av[t,1] <- 1/(1+(exp(a_av[2]+d[t,2])+(exp(a_av[3]+d[t,3]))))

for(k in 2:ne){ # LOOP OVER ENDPOINTS

p_av[t,k] <- p_av[t,1]*exp(a_av[k]+d[t,k])

}

}

# pairwise ORs for all possible pairwise comparisons

# formula: (p1/(1-p1))/(p2/(1-p2))

for (c in 1:(nt-1)) {

for (k in (c+1):nt) {

orR[c,k] <- (p_av[k,1]/(1-p_av[k,1])) / (p_av[c,1]/(1-p_av[c,1])) # response

lorR[c,k] <- log(orR[c,k])

orPR[c,k] <- (p_av[k,2]/(1-p_av[k,2])) / (p_av[c,2]/(1-p_av[c,2])) # partial response

lorPR[c,k] <- log(orPR[c,k])

}

}

for (k in 1:nt){

p_or[k] <-p_av[k,1]+p_av[k,2]

}

# ranking on relative scale

for (k in 1:nt) {

rk[k] <- nt-rank(p_av[,1],k) # CR

rk2[k] <- nt-rank(p_or[],k) # CR+PR

best[k] <- equals(rk[k],1) #calculate probability that treat k is best w.r.t. CR

best2[k] <- equals(rk2[k],1) #calculate probability that treat k is best w.r.t. CR+PR

# calculates probability that treat k is h-th best

for (h in 1:nt){ prob[h,k] <- equals(rk[k],h) }

}

} # *** PROGRAM ENDS

# Data

list(ne=3, ns=17, nt=16, ns1=6)

| r[,1,1] | r[,1,2] | r[,1,3] | r[,2,1] | r[,2,2] | r[,2,3] | n[,1 | n[,2 | Tx[,1] | Tx[,2] | na[ | # Study - Comparison |
| --- | --- | --- | --- | --- | --- | --- | --- | --- | --- | --- | --- |
| 10 | 19 | 95 | 0 | 16 | 94 | 114 | 110 | 1 | 15 | 2 | # GMY302 - OblDex vs Dex |
| 2 | 23 | 101 | 7 | 53 | 313 | 126 | 373 | 1 | 11 | 2 | # OPTIMUM - Thal/ThalDex vs Dex |
| 5 | 51 | 280 | 41 | 80 | 212 | 336 | 333 | 1 | 2 | 2 | # APEX - Bor/BorDex vs Dex |
| 3 | 32 | 141 | 43 | 65 | 69 | 176 | 177 | 1 | 3 | 2 | # MM-009 - LenDex vs Dex |
| 9 | 33 | 133 | 43 | 63 | 70 | 175 | 176 | 1 | 3 | 2 | # MM-010 - LenDex vs Dex |
| 0 | 15 | 138 | 3 | 92 | 207 | 153 | 302 | 1 | 4 | 2 | # MM-003 - PomDex vs Dex |
| 0 | 40 | 24 | 0 | 37 | 30 | 64 | 67 | 2 | 11 | 2 | # Hjorth 2012 - Thal/ThalDex vs Bor/BorDex |
| 25 | 61 | 48 | 56 | 52 | 27 | 134 | 135 | 11 | 12 | 2 | # Garderet 2012 - BorThalDex vs Thal/ThalDex |
| 29 | 261 | 175 | 58 | 298 | 108 | 465 | 464 | 2 | 9 | 2 | # ENDEAVOR - CarDex vs Bor/BorDex |
| 37 | 227 | 132 | 126 | 219 | 51 | 396 | 396 | 3 | 6 | 2 | # ASPIRE - CarLenDex vs LenDex |
| 11 | 128 | 139 | 44 | 187 | 50 | 278 | 281 | 2 | 5 | 2 | # OPTIMISMM - PomBorDex vs Bor/BorDex |
| 60 | 148 | 173 | 107 | 128 | 152 | 381 | 387 | 2 | 10 | 2 | # PANORAMA1 - PanoBorDex vs Bor/BorDex |
| 24 | 189 | 112 | 14 | 238 | 69 | 325 | 321 | 3 | 7 | 2 | # ELOQUENT-2 - EloLenDex vs LenDex |
| 24 | 235 | 103 | 42 | 240 | 78 | 362 | 360 | 3 | 8 | 2 | # Tourmaline-MM1 - IxaLenDex vs LenDex |
| 21 | 127 | 99 | 46 | 153 | 52 | 247 | 251 | 2 | 13 | 2 | # Castor - DaraBorDex vs Bor/BorDex |
| 54 | 162 | 67 | 123 | 143 | 20 | 283 | 286 | 3 | 14 | 2 | # Pollux - DaraLenDex vs LenDex |
| 8 | 125 | 189 | 14 | 130 | 180 | 322 | 324 | 2 | 16 | 2 | # Orlowski - PLDBor vs Bor/BorDex |
| 0 | 19 | 95 | 0 | 16 | 94 | 114 | 110 | 1 | 15 | 2 | # GMY302 - OblDex vs Dex |

END

# Initial values

# Chain 1

list(a=structure(.Data=c( NA, 0, 0, NA, 0, 0, NA, 0, 0, NA, 0, 0, NA, 0, 0, NA, 0, 0, NA, 0, 0, NA, 0, 0,NA, 0, 0, NA, 0, 0, NA, 0, 0, NA, 0, 0, NA, 0, 0, NA, 0, 0, NA, 0, 0, NA, 0, 0, NA, 0, 0),.Dim=c(17,3)), d=structure(.Data=c(NA, NA, NA, NA, 0, 0, NA, 0, 0, NA, 0, 0, NA, 0, 0, NA, 0, 0, NA, 0, 0, NA, 0, , NA, 0, 0, NA, 0, 0, NA, 0, 0, NA, 0, 0, NA, 0, 0, NA, 0, 0, NA, 0, 0, NA, 0, 0),.Dim=c(16,3)))

# Chain 2

list(a=structure(.Data=c(NA, 0, 0, NA, 0, 0, NA, 0, 0, NA, 0, 0, NA, 0, 0, NA, 0, 0, NA, 0, 0, NA, 0, 0, NA, 0, 0, NA, 0, 0, NA, 0, 0, NA, 0, 0, NA, 0, 0, NA, 0, 0, NA, 0, 0, NA, 0, 0, NA, 0, 0),.Dim=c(17,3)),d=structure(.Data=c(NA, NA, NA, NA, 0, 0, NA, 0, 0, NA, 0, 0, NA, 0, 0, NA, 0, 0, NA, 0, 0, NA, 0, 0, NA, 0, 0, NA, 0, 0, NA, 0, 0, NA, 0, 0, NA, 0, 0, NA, 0, 0, NA, 0, 0, NA, 0, 1),.Dim=c(16,3)))

# Chain 3

list(a=structure(.Data=c(NA, 0, 0, NA, 0, 0, NA, 0, 0, NA, 0, 0, NA, 0, 0, NA, 0, 0, NA, 0, 0, NA, 0, 0, NA, 0, 0, NA, 0, 0, NA, 0, 0, NA, 0, 0, NA, 0, 0, NA, 0, 0, NA, 0, 0, NA, 0, 0, NA, 0, 0),.Dim=c(17,3)),d=structure(.Data=c(NA, NA, NA, NA, 0, 0, NA, 1, 0, NA, 0, 0, NA, 1, 0, NA, 0, 0, NA, 1, 0, NA, 0, 0, NA, 1, 0, NA, 0, 0, NA, 1, 0, NA, 0, 0, NA, 1, 0, NA, 0, 0, NA, 1, 0, NA, 0, 0),.Dim=c(16,3)))
