# Supplementary material for: Multinomial network meta-analysis using response rates: relapsed/refractory multiple myeloma treatment rankings differ depending on the choice of outcome
Source: BMC Cancer. 2022 May 30;22:591. doi: 10.1186/s12885-022-09571-8 (PMC9150316; doi:10.1186/s12885-022-09571-8)
Supplement: Supplementary file 2 — Additional file 2: Appendix B. Detailed description and numerical example of multinomial network meta-analysis [file 12885_2022_9571_MOESM2_ESM.docx]

# Appendix B

# Detailed description and

# numerical example of

# multinomial network meta-analysis

As the data contain ordered categorical outcomes, the likelihood which relates the data to the parameters of the models, was specified as $r_{iTk}\sim multinomial(p_{iTk},n_{iT})$. being the probability for response category $k$ in patients with treatment in study , the number of patients within each response category $k$ and the total number of patients with treatment in study .

For modelling ordered categorical outcomes, the fixed effect multinomial logistic model was specified as:

$$\log\left( Y_{iTk} \right)=\left\{ \begin{aligned} \alpha_{ik} \\ \alpha_{ik}+\beta_{ibzk}=\alpha_{ik}+d_{zk}-d_{bk} \end{aligned} | {if \left( T=b \right), b\in1,2,\ldots,tx \atop if \left( T=z \right), z>b, z\in2,3,\ldots,tx} \right. k=2,3$$

$$d_{12}=d_{13}=0$$

Each treatment combination $T$ is designated to a specific index. Therefore, this index runs from 1 to the total number of treatments $tx$. For each study one treatment is assigned as the base treatment $b$ (i.e., often the control arm treatment) and the other treatment as the non-base treatment(i.e., often the experimental arm treatment). The non-base treatments follow all the base treatments $b$ in numerical order and therefore has indices running from 2 to $tx$.

Dexamethasone (treatment index=1) is taken to be the reference treatment for the presented analysis results but note that is not necessarily dexamethasone in every trial. are the hazards for response category $k$ among all patients for treatment *T* in study . The odds are only estimated for category $k=2$ (PR-group) and $k=3$ (<PR-group), because the odds for category $k=1$ (CR-group) is inherent for ordered categorical outcomes. are the log odds for category $k$ for base treatment $b$ in study $i$. are the relative effects for treatment versus , and is estimated for PR and <PR separately. are identified by expressing them in terms of the reference treatment dexamethasone: $\beta_{ibzk}=d_{zk}-d_{bk}$ with $d_{12}=d_{13}=0$.

The log odds were transformed to response specific probabilities for $k=2$ (PR) and $k$=3 (<PR) by the following formula:

$$p_{iTk}=\frac{Y_{iTk}}{1+\sum_{m=2}^{3} Y_{iTm}}$$

The probability of response category CR, corresponding to $k=1$, is inherent and calculated as

$$p_{iT1}=1-\sum_{m=2}^{3} p_{iTm}$$

The remaining $d$’s (one for each treatment) were calculated based on the available direct RCT evidence and the requirement that the indirect evidence should generate similar results.

A smaller network was used to show the step-by-step calculations of the multinomial network meta-analysis. For this network, data from two trials were synthesized:

1. POLLUX (comparing DaraLenDex and LenDex) and
2. ASPIRE (comparing CarLenDex and LenDex).

LenDex is used as reference treatment in this network. In Figure 6 the network is presented with three treatment regimens: [1] LenDex, [2] DaraLenDex and [3] CarLenDex (the numbers between square brackets also correspond to their treatment index in the WinBUGS code file). The solid lines between the treatments indicate direct evidence available from a phase III RCT and the dotted line between the treatments indicate indirect evidence used in the NMA. Every white colored box represents a treatment regimen and is accompanied by a grey box. In the grey boxes the trial data and WinBUGS results are presented. The sources (i.e., POLLUX, ASPIRE or WinBUGS) are followed by three percentages (i.e., CR/PR/<PR rates) and the number of patients (N) assigned to that specific treatment is presented.


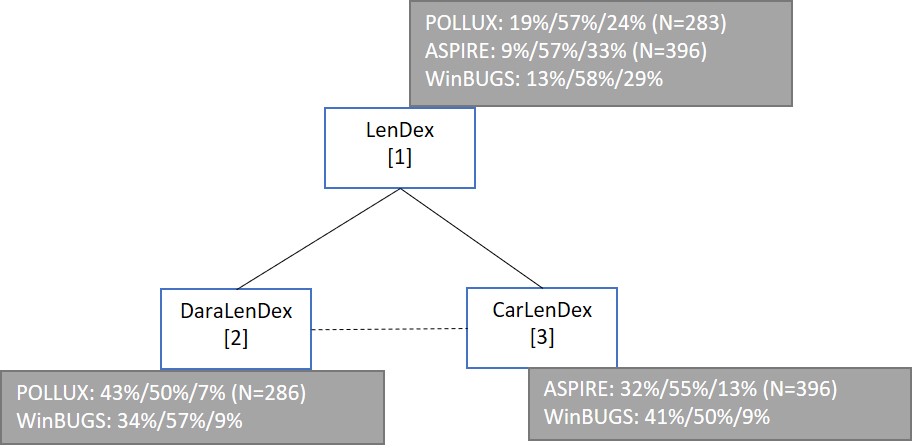


Fig.6 Illustrative example of the network meta-analysis (ASPIRE and POLLUX)

**The data**

In order to populate the WinBUGS data code file, the following variables were used and explained here:

*i*=study index (1=POLLUX, 2=ASPIRE)

*j*=treatment index (1=LenDex, 2=DaraLenDex, 3=CarLenDex)

*k*=outcome index (1=CR, 2=PR, 3=<PR)

*Tx[i,1]* = control treatment index j (1=LenDex, 2=DaraLenDex, 3=CarLenDex)

*Tx[i,2]*=experimental treatment index j (1=LenDex, 2=DaraLenDex, 3=CarLenDex)

na[i] = number of arms in study i (i.e., 2 in this example)

The variable *r[i,j,k]* is the number of patients in study *i* for treatment *j* and outcome *k* (i.e., 1 for CR, 2 for PR and 3 for <PR).

The WinBUGS code for the data is:


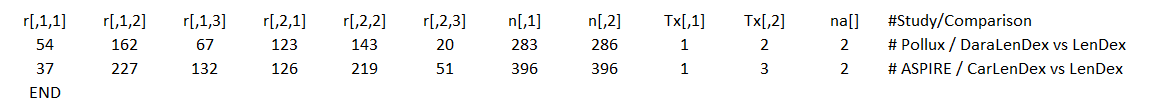


The WinBUGS code for the initial values is:


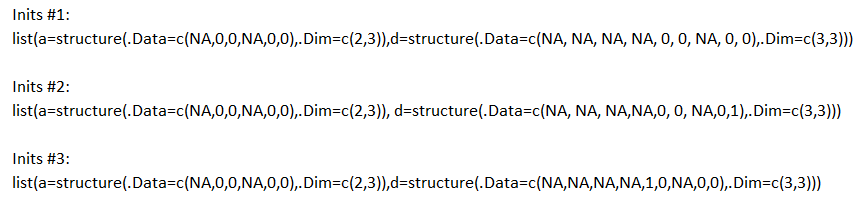


The model estimates based on $r_{iTk}$ which is the number of patients within each response category $k \left( k=2=PR, k=3=<PR \right).$ And of course, the $r_{iT1}$ which is the number of CR patients with treatment in study is calculated using ${1-r}_{iT2}-r_{iT3}$.

The likelihood which relates the data to the parameters of the models, was specified as multinomial distribution with two parameters representing the proportion of PR patients ($p_{iT2}$) or <PR patients ($p_{iT3}$) with treatment in study and the total number of patients with treatment in study : $r_{iTk}\sim multinomial(p_{iTk},n_{iT})$.

And so, the fixed effect ordered categorical model was specified as^12, 33^:


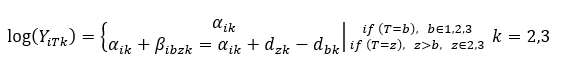


In this smaller network this means that for every iteration the model calculates the $\alpha_{12}$, $\alpha_{13}$ and $d_{22}$,$d_{23}$ for POLLUX and $\alpha_{22}$, $\alpha_{23}$ and $d_{32}$,$d_{33}$ for ASPIRE after drawing new numbers for the different response categories PR and <PR from the multinomial distribution.

The following steps are done in the model for each study and every treatment arm.

Step 1: Calculate all the proportions per response category

Calculate the proportions per response category by dividing the number of patients in the response category by the number of intention-to-treat patients in the specific arm. For example, there were 396 LenDex ITT patients in the ASPIRE trial of which were 37 CR, 227 PR and 132 <PR patients. Therefore, the proportion of CR LenDex ASPIRE patients is $p_{iTk}$ = $p_{211}=\frac{37}{396}=9\%$, PR LenDex patients is $p_{212}=\frac{227}{396}=57\%$ and <PR LenDex patients is $p_{213}=\frac{132}{396}=33\%$.

|  |  | i = 1 = POLLUX | |  | i = 2 = ASPIRE | |
| --- | --- | --- | --- | --- | --- | --- |
|  |  | T =1=LenDex | T=2=DaralenDex |  | T=1=LenDex | T=3=CarLenDex |
|  | response | $p_{11k}$ | $p_{12k}$ |  | $p_{21k}$ | $p_{22k}$ |
| k=1 | CR | 19% | 43% |  | 9% | 32% |
| k=2 | PR | 57% | 50% |  | 57% | 55% |
| k=3 | <PR | 24% | 7% |  | 33% | 13% |

Step 2: Calculate (Y) the hazards of the <PR category

The hazards of the <PR category ($Y_{iT3}$) is calculated using both the proportion of PR and <PR patients calculated in step 1. The formula for $Y_{iT3}$ can be deduced from the formula:

$$p_{iT3}=\frac{Y_{iT3}}{1+\sum_{m=2}^{3} Y_{iTm}}$$

Which gives us

$$Y_{iT3}=\frac{p_{iT3}}{(1-p_{iT2}-p_{iT3})}$$

So, for the LenDex arm in POLLUX the $Y_{113}=\frac{p_{113}}{1-p_{112}-p_{113}}=\frac{24\%}{1-57\%-24\%}=1.24$ and for DaraLenDex $Y_{123}=\frac{p_{123}}{1-p_{122}-p_{123}}=\frac{7\%}{1-50\%-7\%}=0.16$.

|  |  | i = 1 = POLLUX | |  | i = 2 = ASPIRE | |
| --- | --- | --- | --- | --- | --- | --- |
|  |  | T =1=LenDex | T=2=DaralenDex |  | T =1=LenDex | T=3=CarLenDex |
|  | response | $Y_{113}$ | $Y_{123}$ |  | $Y_{213}$ | $Y_{233}$ |
| k=3 | <PR | 1.24 | 0.16 |  | 3.57 | 0.40 |

Step 3: Calculate the hazards (Y) of the PR category

The hazards of the PR category ($Y_{iT2}$) is calculated using both the proportion of PR and the hazards of the <PR category. The formula for $Y_{iT2}$ can be deduced from the formula:

$$p_{iT2}=\frac{Y_{iT2}}{1+\sum_{m=2}^{3} Y_{iTm}}$$

Which gives us

$$Y_{iT2}=\frac{\left( 1+Y_{iT3} \right)p_{iT2}}{1-p_{iT2}}$$

So, for the LenDex arm in POLLUX the $Y_{112}=\frac{(1+Y_{113})p_{112}}{1-p_{112}}=\frac{\left( 1+1.24 \right)*57\%}{1-57\%}=3.00.$

|  |  | i = 1 = POLLUX | |  | i = 2 = ASPIRE | |
| --- | --- | --- | --- | --- | --- | --- |
|  |  | T =1=LenDex | T=2=DaralenDex |  | T =1=LenDex | T=3=CarLenDex |
|  | response | $Y_{112}$ | $Y_{122}$ |  | $Y_{212}$ | $Y_{232}$ |
| k=2 | PR | 3.00 | 1.16 |  | 6.14 | 1.74 |

Step 4: Calculate the log hazards ($\boldsymbol{\alpha}_{\boldsymbol{i}\boldsymbol{2}}$ and $\boldsymbol{\alpha}_{\boldsymbol{i}\boldsymbol{3}}$) for PR and <PR categories

Take the log of the hazards for PR and <PR categories. So, for LenDex in the POLLUX trial the $\alpha_{12}=\ln\left( Y_{112} \right)=\ln\left( 3 \right)=$ 1.10

|  |  | i =1=POLLUX |  | i=2=ASPIRE |
| --- | --- | --- | --- | --- |
|  | response | $\alpha_{1k}$ |  | $\alpha_{2k}$ |
| k=2 | PR | 1.10 |  | 1.81 |
| k=3 | <PR | 0.22 |  | 1.27 |

Step 5: Calculate the relative difference in hazards for PR and <PR categories for the non-reference treatments (d’s)

So, using the following formula we can calculate the d’s:


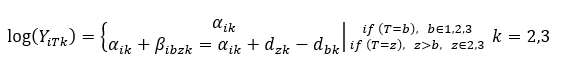


Which gives us

$$d_{ik}=\log\left( Y_{iTk} \right)-\alpha_{ik}$$

Then for DaraLenDex we get for PR $d_{12}=\log\left( Y_{122} \right)-\alpha_{12}=\log\left( 3.00 \right)-1.10=-0.95$.

|  |  | i =1=POLLUX |  | i=2=ASPIRE |
| --- | --- | --- | --- | --- |
|  | response | $d_{1k}$ |  | $d_{2k}$ |
| k=2 | PR | -0.95 |  | -1.26 |
| k=3 | <PR | -2.03 |  | -2.18 |

Step 6: Calculate the proportion of patients for the reference treatment (LenDex) combining LenDex data from both trials

The model uses the average of the proportions of LenDex for PR and <PR. This means for PR $\alpha_{avg,2}=average\left( \alpha_{12},\alpha_{22} \right)=\frac{(1.10+1.81)}{2}=1.46$ and <PR $\alpha_{avg,3}=average\left( \alpha_{13},\alpha_{23} \right)=\frac{\left( 0.21, 1.27 \right)}{2}=0.74$.

Step 7: Calculate the proportion of CR patients

$$\pi_{T1}=\frac{1}{1+exp(\alpha_{avg,2}+d_{T2})+exp(\alpha_{avg,3}+d_{T3})}$$

Therefore, for LenDex the proportion of CR patients becomes

$$\pi_{11}=\frac{1}{1+\exp\left( 1.46+0 \right)+exp(0.74+0)}=13\%$$

The CR proportions for DaraLendex and CarLendex are presented in the table below.

| Response | LenDex | DaraLenDex | CarLenDex |
| --- | --- | --- | --- |
| CR | 13% | 34% | 41% |

Step 7: Calculate the proportion of PR and <PR patients

$$\pi_{Tk}=\pi_{T1}*\exp\left( \alpha_{avg,k}+d_{Tk} \right), k=2,3$$

Subsequently, for LenDex the proportion of PR patients is

$$\pi_{12}=\pi_{11}*\exp\left( \alpha_{avg,2}+d_{12} \right)=13\%*\exp\left( 1.46+0 \right)=58\%$$

For all the other proportions please see table below.

| Response | LenDex | DaraLenDex | CarLenDex |
| --- | --- | --- | --- |
| PR | 58% | 57% | 50% |
| <PR | 28% | 9% | 10% |
