# Supplementary material for: Multinomial network meta-analysis using response rates: relapsed/refractory multiple myeloma treatment rankings differ depending on the choice of outcome
Source: BMC Cancer. 2022 May 30;22:591. doi: 10.1186/s12885-022-09571-8 (PMC9150316; doi:10.1186/s12885-022-09571-8)
Supplement: Supplementary file 3 — Additional file 3: Appendix C. SUCRA results [file 12885_2022_9571_MOESM3_ESM.docx]

# Appendix C

# SUCRA results

This appendix presents all SUCRA related results of the multinomial network meta-analysis conducted on response in relapsed and/or refractory multiple myeloma. (see figures 7 to 12).

Abbreviations used in the following figures:

Bor=bortezomib,

Car=carfilzomib,

Dara=daratumumab.

Dex=dexamethasone,

Elo=elotuzumab,

Ixa=ixazomib,

Len=lenalidomide,

Obl=oblimersen,

Pano=panobinostat,

PLD=pegylated liposomal doxorubicin,

Pom=pomalidomide,

Thal=thalidomide


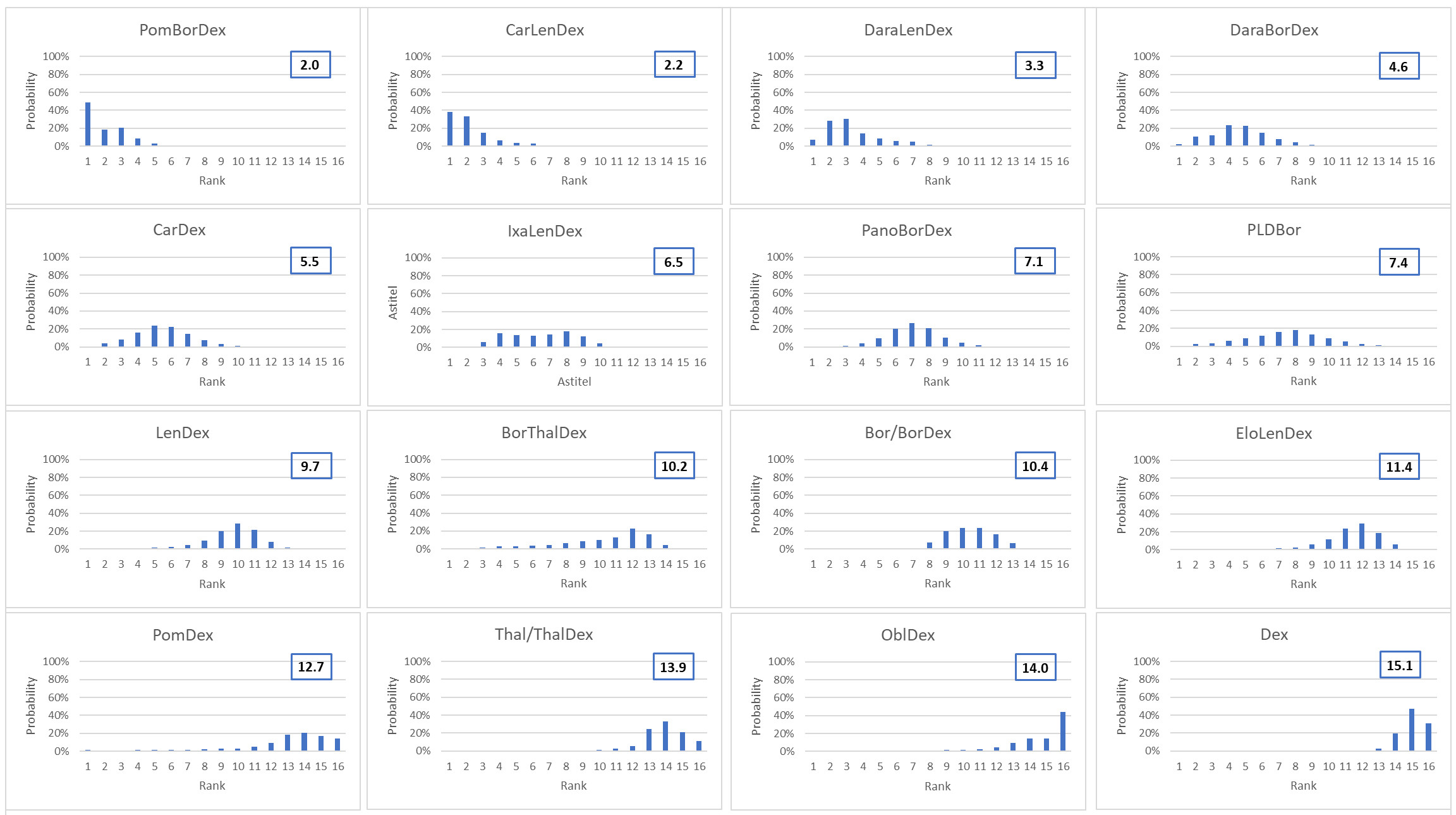
**Fig.7 Distribution of the probabilities of being at each rank for CRR**

Figure 7 presents for every treatment the probabilities of being at each rank (from 1 as best to 16 as worst) are shown together with their mean ranks for CRR.


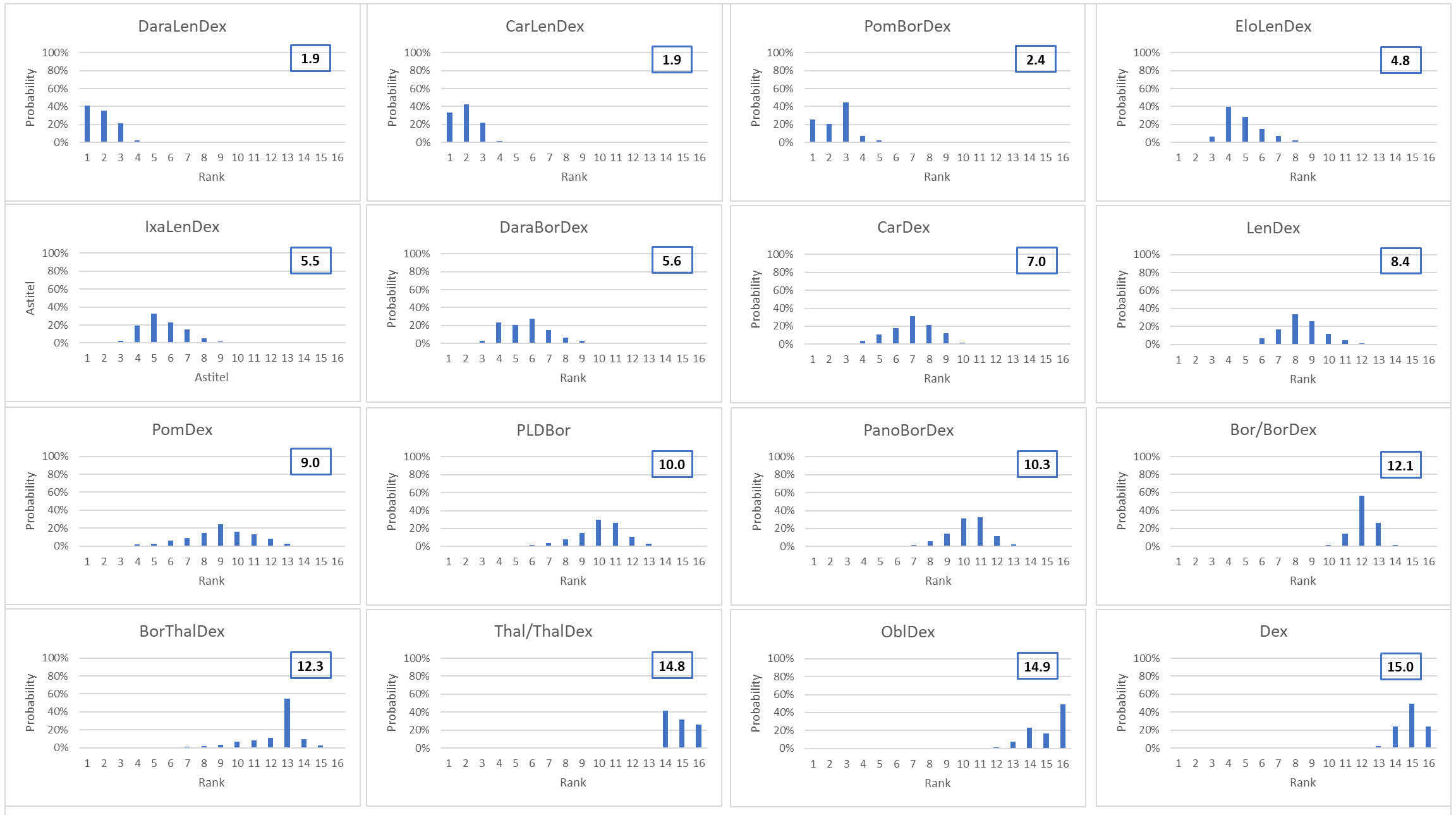
**Fig.8 Distribution of the probabilities of being at each rank for ORR**

Figure 8 presents for every treatment the probabilities of being at each rank (from 1 as best to 16 as worst) are shown together with their mean ranks for ORR.


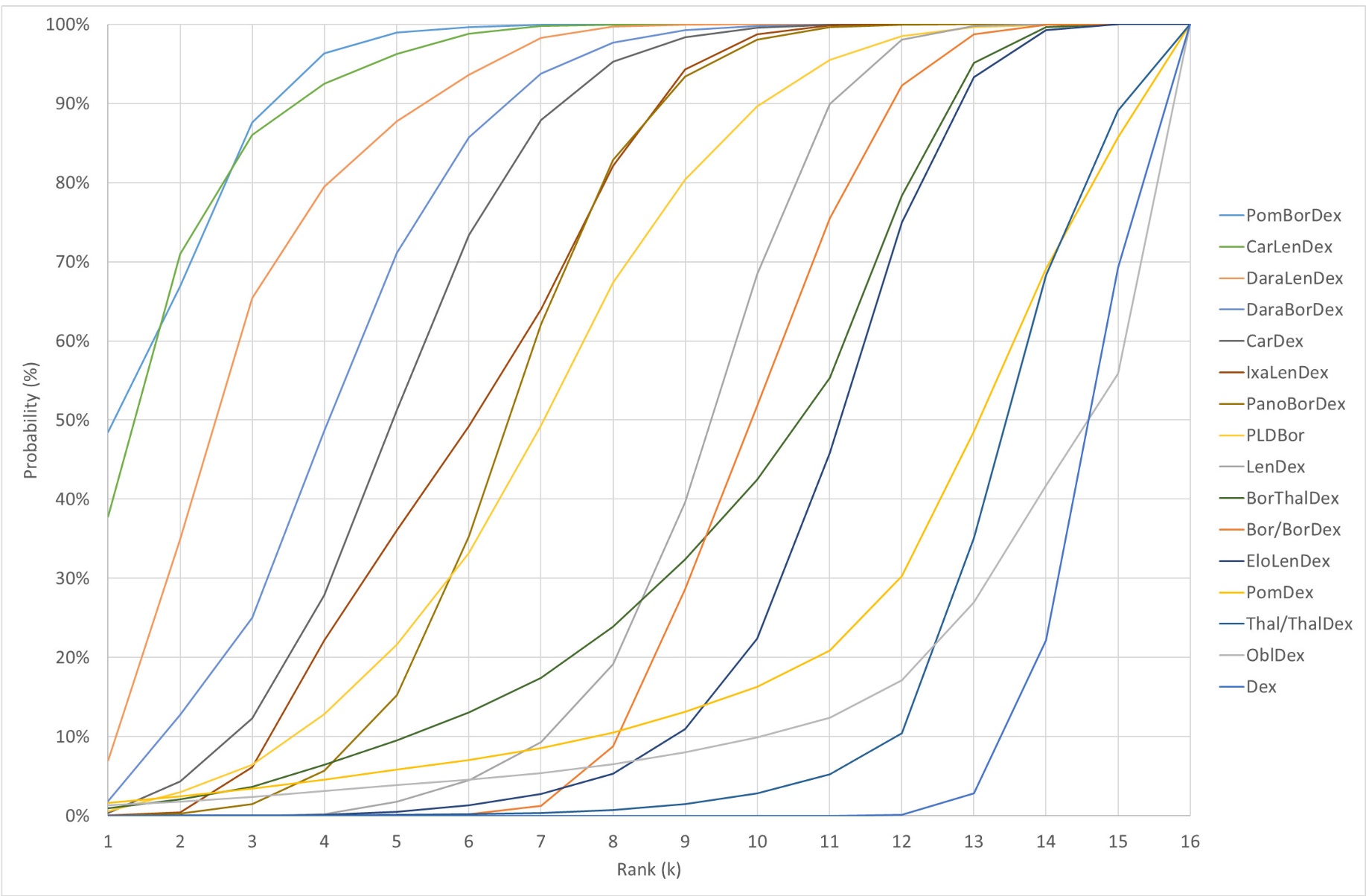


**Fig.9 SUCRA for CRR**

Figure 9 shows the cumulative probability of being among the *k* best treatments for CRR. The surface under the cumulative ranking (SUCRA) curve represents the SUCRA score. The treatments in the legends box are sorted on their SUCRA score.


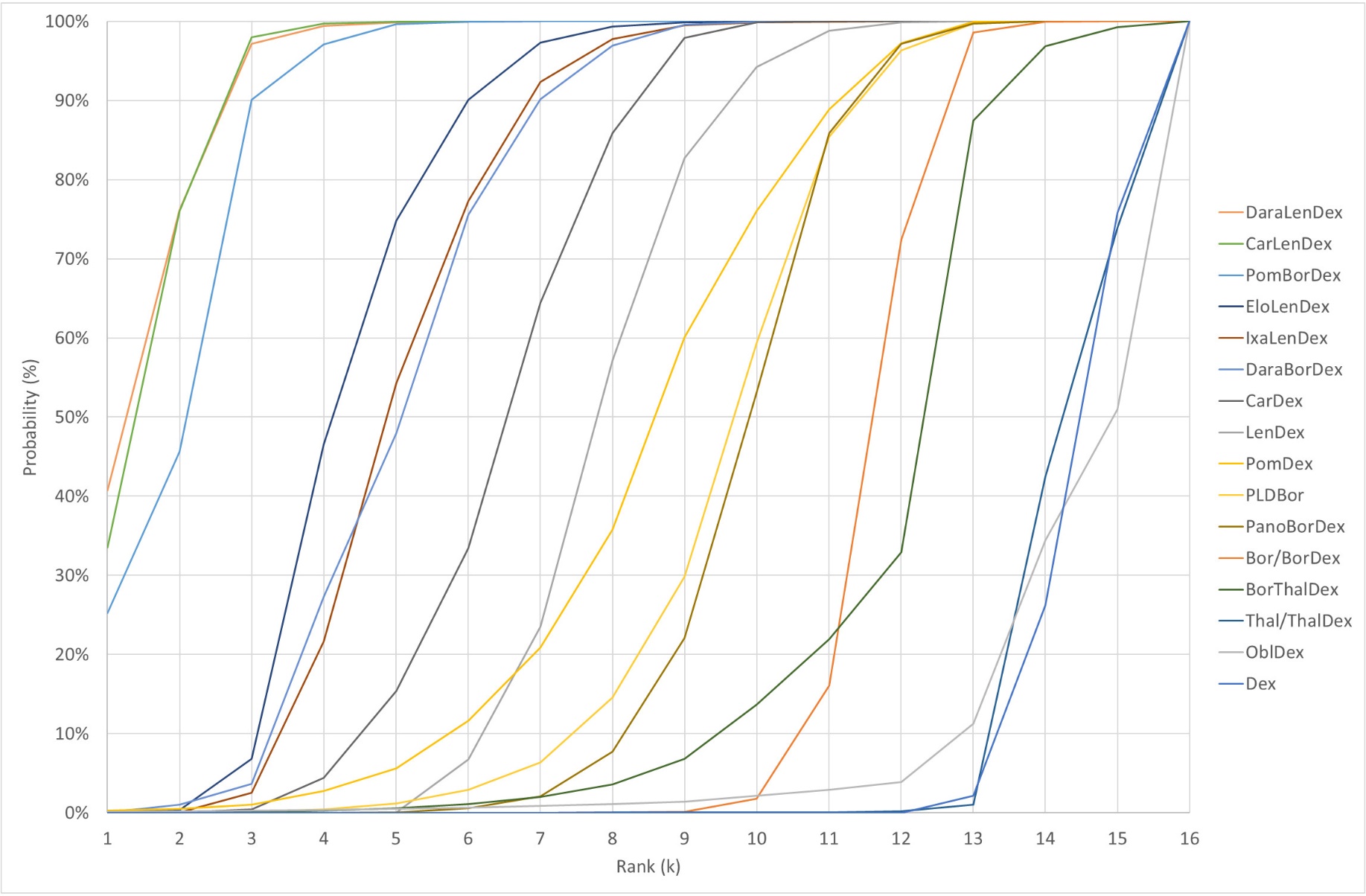


**Fig.10 SUCRA for ORR**

Figure 10 shows the cumulative probability of being among the *k* best treatments for ORR. The surface under the cumulative ranking (SUCRA) curve represents the SUCRA score. The treatments in the legends box are sorted on their SUCRA score.


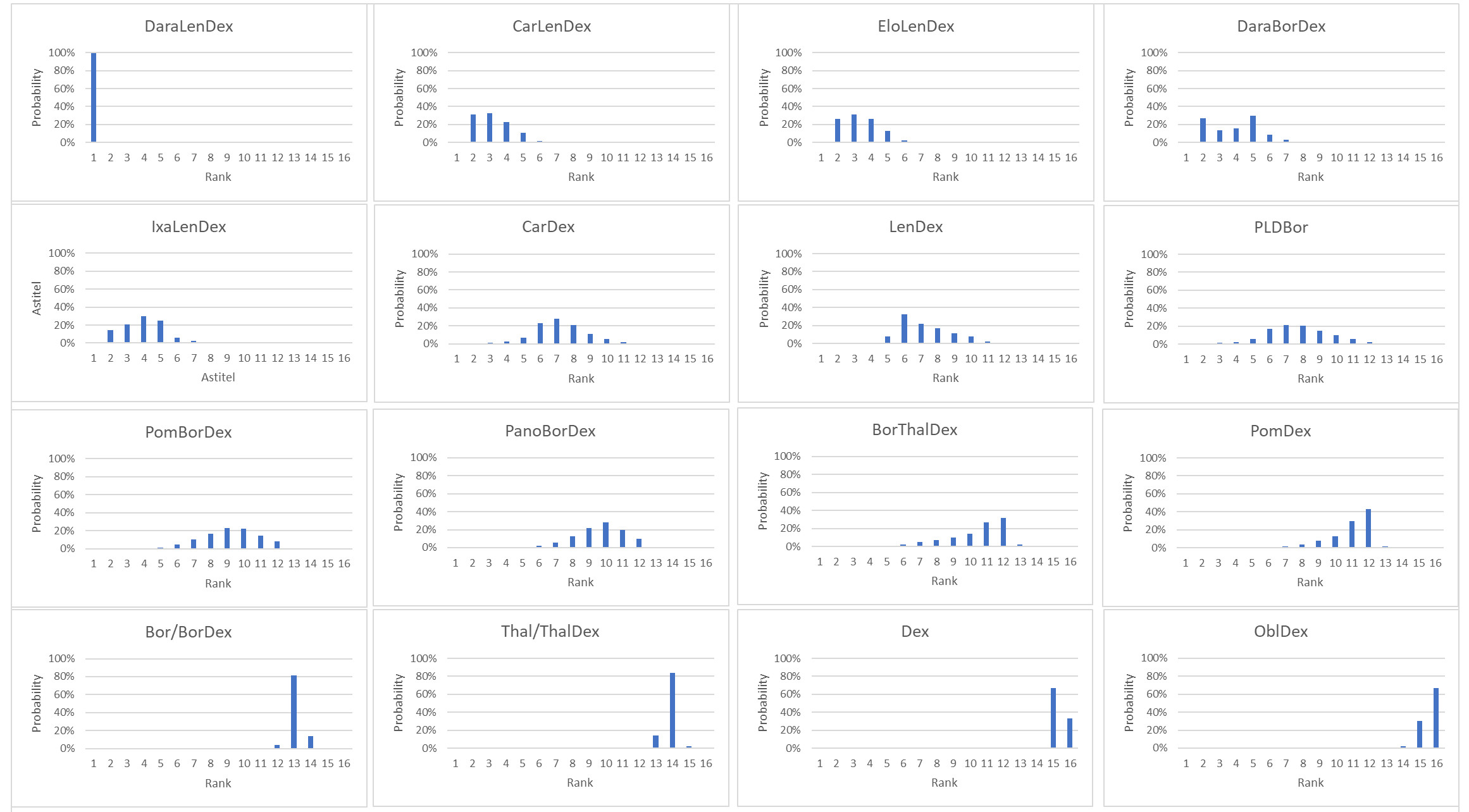


**Fig.11 Distribution of the probabilities of being at each rank for PFS HR**

Figure 11 presents for every treatment the probabilities of being at each rank (from 1 as best to 16 as worst) are shown together with their mean ranks for progression-free survival.


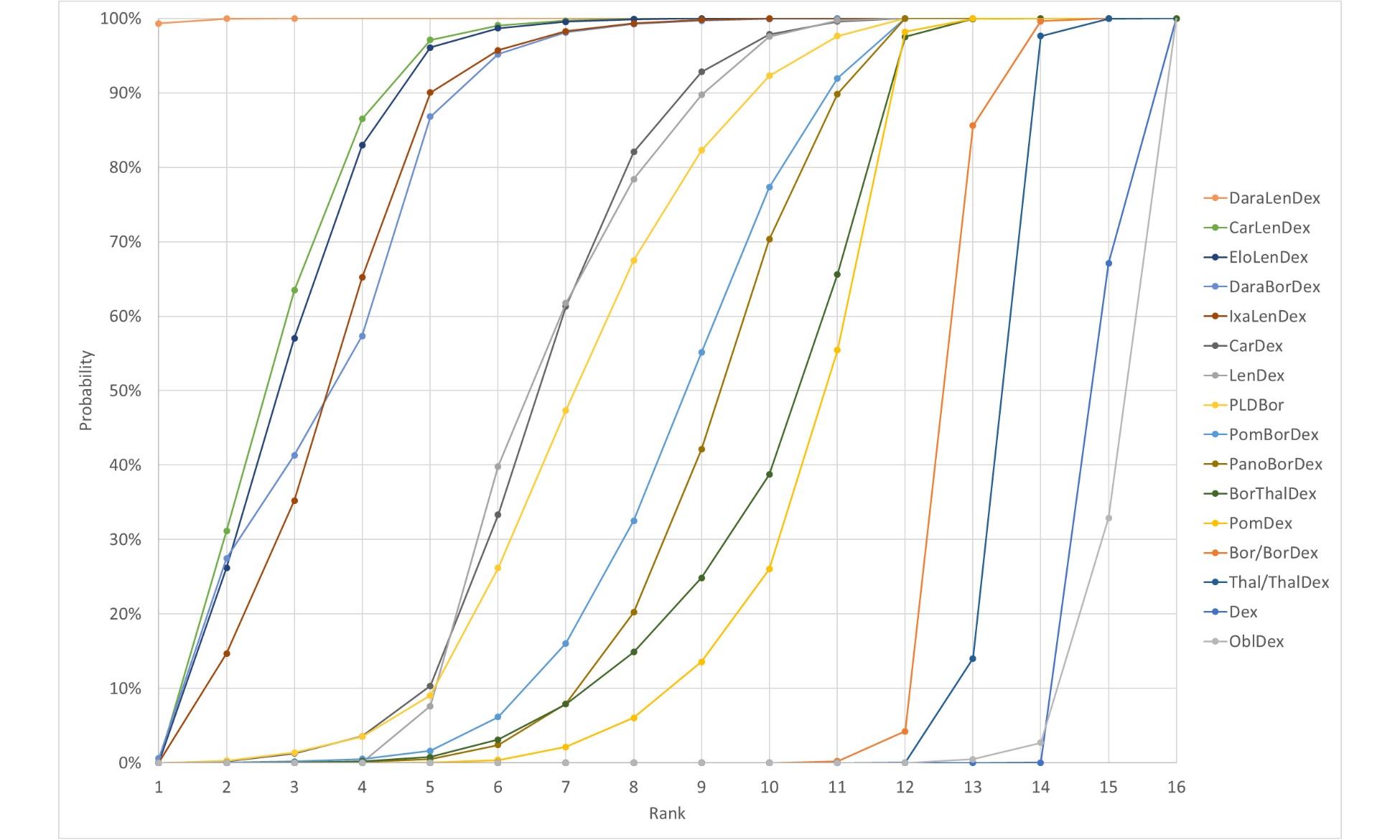


**Fig.12 SUCRA for PFS HR**

Figure 12 shows the cumulative probability of being among the *k* best treatments for progression-free survival. The surface under the cumulative ranking (SUCRA) curve represents the SUCRA score. The treatments in the legends box are sorted on their SUCRA score.
