# Supplementary material for: Multinomial network meta-analysis using response rates: relapsed/refractory multiple myeloma treatment rankings differ depending on the choice of outcome
Source: BMC Cancer. 2022 May 30;22:591. doi: 10.1186/s12885-022-09571-8 (PMC9150316; doi:10.1186/s12885-022-09571-8)
Supplement: Supplementary file 4 — Additional file 4: Appendix D. Results scenario analyses [file 12885_2022_9571_MOESM4_ESM.docx]

# Appendix D

# Results scenario analyses

This appendix presents the scenario analyses on the multinomial NMA in RR MM treatments. Figure 13 presents the forest plots of the three scenario analyses with CRR outcome. Figure 14 shows the three scenario analyses results with ORR outcome.


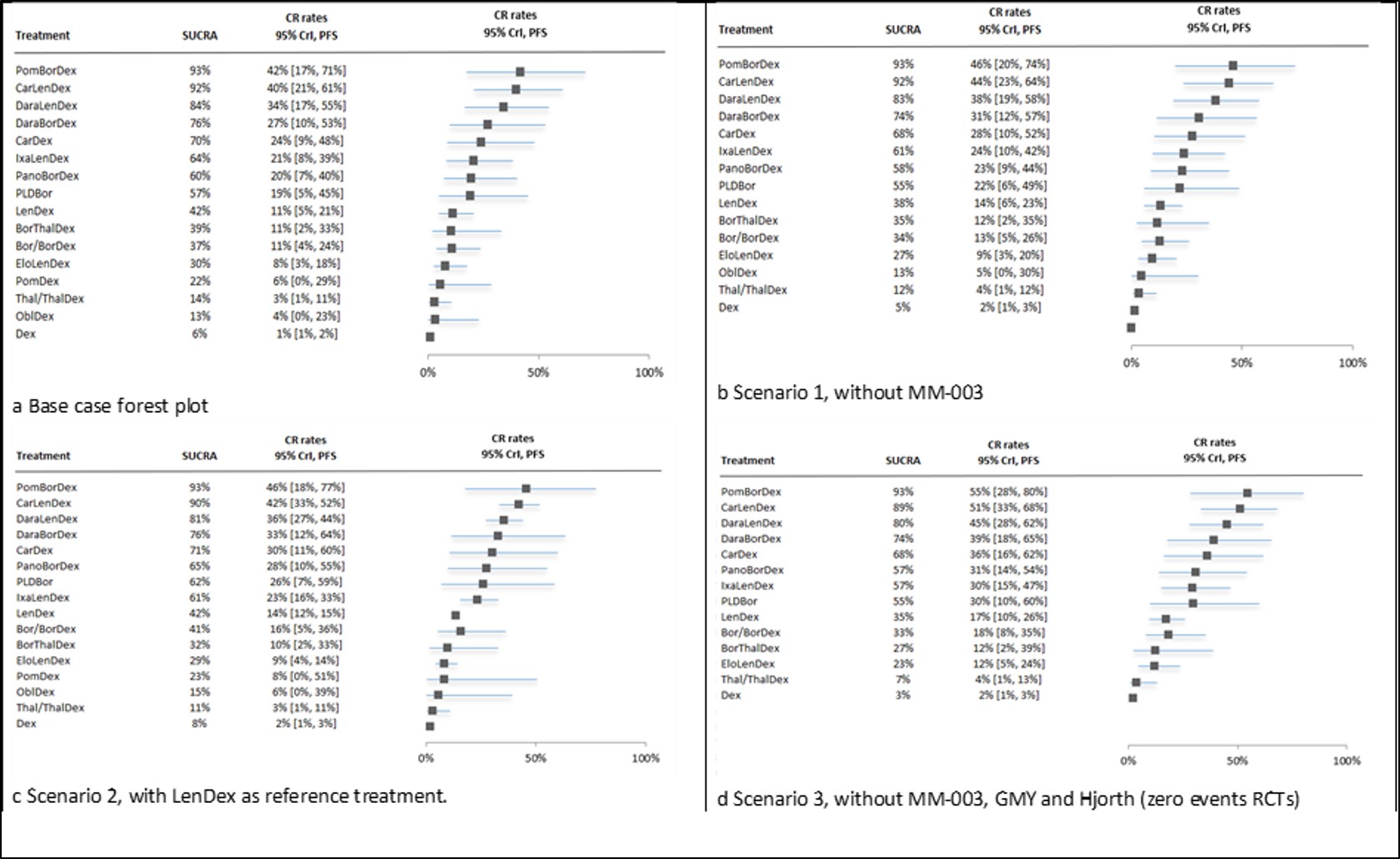
**Fig.13 Forest plots for base case and scenario analysis with CRR outcome.** a Base case CR rates (16 treatments, 17 trials), b scenario 1, without MM-003 (15 treatments, 16 trials), c scenario 2, with LenDex as reference treatment instead of Dex (16 treatments, 17 trials), d scenario 4, without MM-003 trials, GMY and Hjorth 2012 because of zero events (14 treatments, 14 trials).


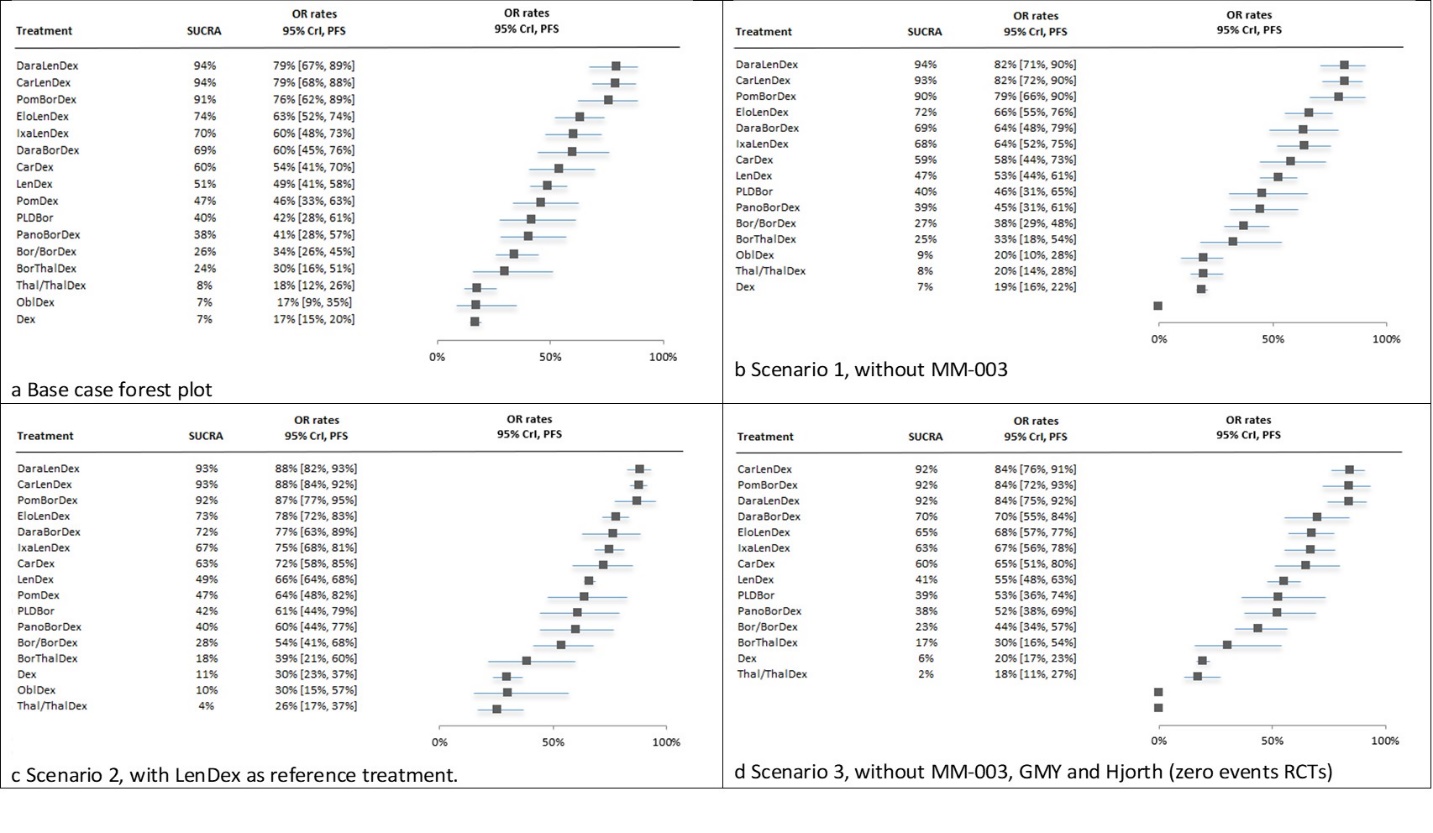
**Fig.14 Forest plots for base case and scenario analysis with ORR outcome.** a Base case OR rates (16 treatments, 17 trials), b scenario 1, without MM-003 (15 treatments, 16 trials), c scenario 2, with LenDex as reference treatment instead of Dex (16 treatments, 17 trials), d scenario 4, without MM-003 trials, GMY and Hjorth 2012 because of zero events (14 treatments, 14 trials).
